# Qishen Granule (QSG) Inhibits Monocytes Released From the Spleen and Protect Myocardial Function via the TLR4-MyD88-NF-κB p65 Pathway in Heart Failure Mice

**DOI:** 10.3389/fphar.2022.850187

**Published:** 2022-03-15

**Authors:** Yanqin Li, Xuan Li, Xu Chen, Xiaoqian Sun, Xiangning Liu, Gang Wang, Yizhou Liu, Lingwen Cui, Tianhua Liu, Wei Wang, Yong Wang, Chun Li

**Affiliations:** ^1^ College of Chinese Medicine, Beijing University of Chinese Medicine, Beijing, China; ^2^ School of Chinese Materia Medica, Beijing University of Chinese Medicine, Beijing, China; ^3^ Beijing Key Laboratory of TCM Syndrome and Formula, Beijing University of Chinese Medicine, Beijing, China; ^4^ School of Life Sciences, Beijing University of Chinese Medicine, Beijing, China; ^5^ Modern Research Center for Traditional Chinese Medicine (TCM), Beijing University of Chinese Medicine, Beijing, China

**Keywords:** heart failure, macrophages, inflammation, splenic monocytes, qishen granule

## Abstract

Preliminary clinical and basic researches have proved that Qishen granule (QSG) is an effective prescription for treating heart failure (HF) in China, with a characteristic of regulating the ratio of M1/M2 macrophage in the myocardium. However, the regulative mechanism of monocytes targeting the cardio-splenic axis has not been fully elucidated. This study aimed to investigate the effects and mechanism of QSG inhibiting the release of splenic monocytes and the recruitment of myocardial tissue both *in vivo* and *in vitro*. Experiments in mice with acute myocardial infarction (AMI)-induced HF demonstrated that QSG could exert anti-inflammatory effects by inhibiting splenic monocytes release and phenotypic changes. Moreover, *in vitro* experiments indicated QSG could inhibit LPS-stimulated macrophage-conditioned medium (CM)-induced H9C2 cardiomyocyte injury by upregulating the key proteins in TLR4-MyD88-NF-κB p65 pathway. In addition, knockdown or overexpression of TLR4 in H9C2 cells further confirmed that QSG could attenuate inflammatory injury in cardiomyocytes via the TLR4-MyD88-NF-κB p65 pathway. Overall, these data suggested that QSG could improve cardiac function and reduce the inflammatory response in AMI-induced HF by inhibiting splenic monocytes release, and protecting myocardial function via the TLR4-MyD88-NF-κB pathway in heart failure mice.

## Introduction

Myocardial infarction is one of the most common and important causes of heart failure (HF). Accumulating evidence showed that post-infarction HF could increase the risk of short- and long-term adverse events in patients with the rising incidence (Bahit et al., 2018; Kim et al., 2020). Although significant progress has been made in the treatment of HF in recent years, the quality of life of patients with HF is still unsatisfying (Willerson, 2019; Baman and Ahmad, 2020). Thus, the new therapeutic targets as well as interventions are essential to improve the treatment of HF.

The latest basic and clinical studies have shown that the inflammation induced by crosstalk between the heart and the spleen (the so-called cardio-splenic axis) dominates the inflammatory injury during the HF process (Jahng et al., 2016; Dunford et al., 2017; Saito et al., 2020). The activation of monocytes in the spleen is the critical resource of myocardial inflammation in HF (Ismahil et al., 2014). Splenic monocytes are generally divided into two monocyte subpopulations, Ly6C^high^ and Ly6C^low^ (Kim et al., 2014; Dutta and Nahrendorf, 2015). In the acute phase of myocardial injury, the spleen mobilizes its Ly6C^high^-led monocytes and releases them into the injury zone (Nahrendorf et al., 2010; Tian et al., 2016), and subsequently activated M1 macrophages to exert pro-inflammatory and phagocytic functions. In the HF process, monocytes/macrophages behave as pro-inflammatory M1 macrophages (Mouton et al., 2020). Thus, inhibiting inflammation and polarization of macrophages to M1 may be a promising treatment option for HF (Wallert et al., 2019; Heimerl et al., 2020).

Toll-like receptors (TLRs) as an important component of the innate immune system are pattern recognition receptors (PRRs) (Ochando et al., 2019). As a member of the TLRs, Toll-like receptor 4 (TLR4) could regulate the inflammatory response of the myocardium. Additionally, the inflammatory signaling pathway mediated by TLR4 plays a crucial role in myocarditis, myocardial infarction, and ischemia-reperfusion injury (Wang et al., 2019; Zhao et al., 2019; Ciesielska et al., 2021). Several studies have shown that the TLR4-MyD88-NF-κB p65 pathway initiates inflammatory responses in spleen and myocardial tissue, which in turn leads to an aggravation of inflammatory injury in myocardial tissue (Wu et al., 2018; Wang X. et al., 2020; Xu et al., 2020). Moreover, TLR4 inhibitor was shown to reduce Ly6C^high^ and CD11b^+^ monocytes in spleen and peripheral blood (Wu et al., 2017), which is considered the most attractive therapeutic strategy for anti-inflammation in HF patients.

As a clinically approved traditional Chinese medicine for treating HF, QSG has been researched for many years (Wang et al., 2017). QSG is derived from the classical formulas Zhengwu Tang and Si Miao Yong An Tang, which are composed of six botanical drugs including *Astragalus camptoceras* Bunge (*Fabaceae*), *Salvia miltiorrhiza* Bunge (*Lamiaceae*), *Lonicera japonica* Thunb. (*Caprifoliaceae*), *Scrophularia ningpoensis* Hemsl. (*Scrophulariaceae*), *Aconitum carmichaelii* Debeaux (*Ranunculaceae*) and *Glycyrrhiza uralensis* Fisch. ex DC. (*Fabaceae*) (30: 15: 10: 10: 9: 6) (Chen et al., 2022). The preparation process and composition identification of QSG were described in detail in our previous study (Wang et al., 2012; Xia et al., 2017; Gao et al., 2020). Our preliminary findings suggested that QSG could exert anti-inflammatory and anti-fibrotic effects by modulating the macrophage phenotype (Lu et al., 2019). To date, how it regulates the macrophage phenotype has not been fully elucidated yet. This study is to systematically explore the protective effect of QSG on the spleen and heart by utilizing left anterior descending (LAD) ligation to prepare HF mice model and RAW 264.7 macrophages conditional supernatant cell model. Furthermore, the effect of QSG on subpopulations changes of monocytes in the cardio-splenic axis and the potential mechanism of anti-inflammatory effects through the TLR4-MyD88-NF-κB p65 pathway were investigated. It will provide an alternative anti-inflammatory therapy for the treatment of HF.

## Materials and Methods

### Drugs

Several herbs in QSG were purchased from Beijing Tong Ren Tang Pharmaceutical Co., Ltd. and processed by the Beijing University of Chinese Medicine. QSG used in this experiment were the same batch as in a published related study (Chen et al., 2021). Fosinopril sodium tablets (fosinopril) were indicated to be effective for patients with HF and used as the positive drug (H19980197, Bristol-Myers Squibb Pharmaceutical Co., Ltd., China).

### Reagents

LPS (l2880, Biodee, China) was used to induce macrophages inflammatory, purchased from Beijing BioDee Biotechnology Co., Ltd. 0.9% normal saline (b020, Jiancheng, Nanjing, China) purchased from Beijing BioDee Biotechnology Co., Ltd.

### Animals and Ethics Statement

All animal experimental protocols in this study were approved by the Animal Ethics Committee of Beijing University of Chinese Medicine (approval number “BUCM-4-2021113003-4081”) and conformed to the “Guidelines for the Care and Use of Laboratory Animals” published by the National Institute of Health (NIH Publication No. Resolution No. 85–23, revised in 1996). Fifty male SPF-grade ICR mice (28 ± 2 g) were purchased from Beijing Spefo Technology Co., Ltd. Mice were housed in the animal house of Beijing University of Chinese Medicine (temperature 23 ± 2°C, humidity 50 ± 5%, 12:12 light: dark cycles), fed with conventional feed and water ad libitum. After 3 d of adaptive feeding, the ligation of the mouse left anterior descending branch (LAD) to induce heart failure model as described in our previous study (Li et al., 2016). Briefly, 50 ICR mice underwent left-sided open-heart surgery between the third and fourth rib gaps. After exposing the heart tissue, the LAD was ligated using a 7-0 sterile suture 1–1.5 mm below the left auricle. Mice in the sham-operated group underwent open-chest and threading surgery at the same location in the heart only. Ten mice undergoing LAD ligation were randomly selected for splenectomy, using a previously published method (Tian et al., 2015). A midline dissection of approximately 1 cm was made, the hepatic hilum was clamped, the spleen was removed, and the muscle and skin were sutured with 5-0 sterile suture.

After the mice were awakened, the surviving 43 mice were randomly divided into five groups: 8 in the sham-operated group, 9 in the model group, 9 in the splenectomy group, 9 in the QSG group, and 8 in the fosinopril group. The dosage of QSG is determined according to the equal conversion of clinical effective dose and adjusted according to previous experiments (Lu et al., 2019). After 24 h of the LAD ligation, QSG (dose 5.66 g/kg) at a concentration of 566 mg/ml dissolved in saline was given by gavage according to the bodyweight of 0.1 ml/10 g for 7d in QSG group. And fosinopril (dose 10 mg/kg) at a concentration of 1 mg/ml dissolved in saline was given by gavage according to the bodyweight of 0.1 ml/10 g for 7d in fosinopril group. The sham, model, and splenectomy groups were given the same volume of 0.9% normal saline. Keep the same time of gavage every day, and continue the administration for 7 d. All operations were performed under pentobarbital sodium anesthesia to cut the pain.

### Echocardiographic Evaluation of Cardiac Function

As mentioned before (Lu et al., 2019), using the Vevo 2100 ultrasound system (Vevo TM 2100, Visual Sonics, Canada), mice undergo transthoracic two-dimensional M-mode echocardiography to assess cardiac function.

### Histological Examination Staining

Heart and spleen tissues were fixed in 4% paraformaldehyde, embedded in paraffin, and sectioned at 5 μm. Sections were stained with hematoxylin-eosin (HE) to assess the underlying structure and degree of inflammatory infiltration.

### Immunofluorescence Staining

Paraffin sections of spleen sections were dewaxed, and blocked with serum for 30 min. The sections were incubated overnight at 4°C with anti-CD11b antibody (ab184308; Abcam, United States) in a wet box. Then after being washed with PBS for three times, Alexa Fluor 488 (D001-34, Abcam, United States) was incubated for 60 min at room temperature away from light. Cell nuclei were stained with DAPI (C0065, Solarbio, China).

H9C2 cells were seeded in confocal dishes, after stimulated administration, fixed with 4% paraformaldehyde for 15 min, and permeabilized with 0.5% TritonX-100 for 20 min. Then cells were blocked with 1% BSA in the 37 °C incubator for 1 h. Incubation with anti-Phospho-IκBα (Ser32) antibody (14D4, Cell Signaling Technology, Germany) at 4°C overnight, followed by incubation with the Goat Anti-Rabbit IgG (H + L) Alexa Fluor 488 (AB0141, Abways, China) for 1 h at room temperature away from light. Then Nuclei were stained with DAPI for 5 min.

### CK-MB and LDH Detection

Blood was collected from mice via the abdominal aorta and centrifuged at 3000 rpm/min for 10 min at 4°C after 2 h. The serum supernatant was extracted and stored at −80°C in a freezer. The levels of creatine kinase-MB (CK-MB) (sea479mu, Cloud-clone, China) and lactate dehydrogenase (LDH) (seb864ra, Cloud-clone, China) in the serum were measured using ELISA kit.

### Flow Cytometry Detection

Flow cytometry was used to detect spleen monocytes, blood monocytes and cardiac macrophages. The spleen, blood, and heart were extracted according to the method steps (Lu et al., 2019), flow-related cell populations were detected, and the data were processed using Flow Jo flow analysis software. The flow antibodies used in this experiment included: PE/Cy7^®^ Anti-CD11b antibody (ab218786, Abcam, United States), PE Anti-Ly6c (ab25572, Abcam, United States), Alexa Fluor^®^ 647 Anti-F4/80 antibody (ab 204467, Abcam, United States), FITC Anti-CD11c antibody (ab210308, Abcam, United States).

### Cell Culture

The RAW264.7 macrophages and rat cardiac H9C2 cells used in this study were obtained from the Chinese Cell Line Resource Infrastructure and cultured in DMEM (11995065, Gibco, United States) with 10% FBS (10099141, Gibco, United States) and 1% penicillin/streptomycin (P/S) (15140122, Invitrogen, United States) at 37°C under a humidified atmosphere of 5% CO2. To screen for non-toxic concentrations of QSG, H9C2 and RAW264.7 cells were seeded in 96-well plates and incubated with QSG (1–1,500 μg/ml) for 24 h. Cell viability was then determined using the CCK-8 assay kit (g021-2-1, Jiancheng, Nanjing, China) according to the manufacturer’s instructions. To investigate the effective concentration of QSG in macrophages, RAW264.7 cells were cultured to 80%–90% confluence in a 96-well plate. After different treatments, the cell supernatant was extracted and assayed according to the NO assay kit method (bn27106-500, Biorigin, China). After adding the standards/samples, Griess Reagent I and Griess Reagent II, the plate was mixed well and incubated at room temperature for 5 min. After the reaction is completed, NO concentration was measured at 540 nm using a microplate reader.

To assess the effect of QSG on LPS-induced macrophages, RAW264.7 cells were exposed to LPS (1 μg/ml) for 24 h with/without QSG. Then cell supernatants of conditioned media were collected for further experiments. To investigate the effect of QSG on conditioned medium (CM)-stimulated cardiomyocytes, H9C2 cells were pretreated with QSG for 6 h, and then stimulated with CM (with/without QSG) for 24 h.

### Knockdown of TLR4 With siRNA

TLR4 siRNA (A10001, GenePharma, China) was used to inhibit TLR4 expression according to the manufacturer’s instructions. The sequences (5′-3′) are GCA​GCA​GGU​CGA​AUU​GUA​UTT; AUA​CAA​UUC​GAC​CUG​CUG​CTT. Briefly, H9C2 cells were plated into 6-well plates for 24 h, and the medium was replaced with Opti-MEM (31985070, Gibco, United States) 2 h before transfection. Subsequently, cells were transfected with 8 μl RNA oligo (Tlr4-rat-2571 or negative control) and 8 μl GP-transfect-Mate (G04009, GenePharma, China)/well for 6 h, followed by DMEM (10% FBS; no P/S) for 18 h instead. Proteins were extracted to check knockdown efficiency at 48 h after the transfection.

### Overexpression of TLR4 Plasmid

Following the manufacturer’s instructions, use rTlr4 pcDNA3.1-T2A-DsRed (211208PC01, Hanbio, China) to overexpress TLR4. In short, H9C2 cells were plated into 6 mm plates for 24 h, and the medium was replaced with Opti-MEM 2 h before transfection. And cells were transfected with 8 μg plasmid (rTlr4 pcDNA3.1-T2A-DsRed or pcDNA3.1-T2A-DsRed) and 20 μL LipoFiterTM 3.0 (HB-TLRF3-1,000, Hanbio, China)/well for 6 h. Then, the medium was replaced with DMEM (10% FBS; no P/S). After 48 h, immunofluorescence verification was performed, and proteins were extracted from cells for western blot analysis.

### Western Blot Analysis

Western blot analysis was used to detect protein expression levels in heart tissues or cells. Briefly, heart tissues or cells were lysed in pre-cold RIPA buffer (c1053-100, APPLYGEN, China) with a 0.5% protease inhibitor (p1625, APPLYGEN, China) and 1% phosphatase inhibitor (p1260-1, APPLYGEN, China). The protein concentration of each sample was measured using a BCA kit (P1511-1, APPLYGEN, China). Equal quantities of the proteins from each group were loaded on 10% PAGE gel fast preparation kit (PG112, Epizyme, China), electrophoresed, and transferred onto PVDF membranes (p2120-6, APPLYGEN, China). The membranes were incubated with primary and secondary antibodies and then treated with ECL (SQ201, Epizyme, China). The following antibodies were used: anti-TLR4 (19811-1-AP, Proteintech, United States), anti-MyD88 (D80F5, Cell Signaling Technology, Germany), Phospho-IκBα (Ser32) (14D4, Cell Signaling Technology, Germany), anti-IKBα (ab32518, Abcam, United States), Phospho-NF-κB p65 (Ser S536) (93H1, Cell Signaling Technology, Germany), anti-NF-κB p65 (D14E12, Cell Signaling Technology, Germany), and anti-GAPDH (ab8246, Abcam, United States), anti-mouse IgG H&L (AB0102; Abways, China) and anti-rabbit IgG H&L (AB0141; Abways, China). Finally, Image Lab software was used for band gray analysis.

### Statistical Analysis

GraphPad Prism 7.0 statistical software was used for data processing and analysis, and the results obtained were expressed as mean ± standard error (x ± SEM). The *t*-test and one-way analysis of variance were used to compare the differences between two or more groups with statistical significance. *p* < 0.05 indicates that the difference is statistically significant.

## Experimental Results

### Effects of QSG on Cardiac Function in Mice With AMI-Induced HF

**FIGURE 1:**
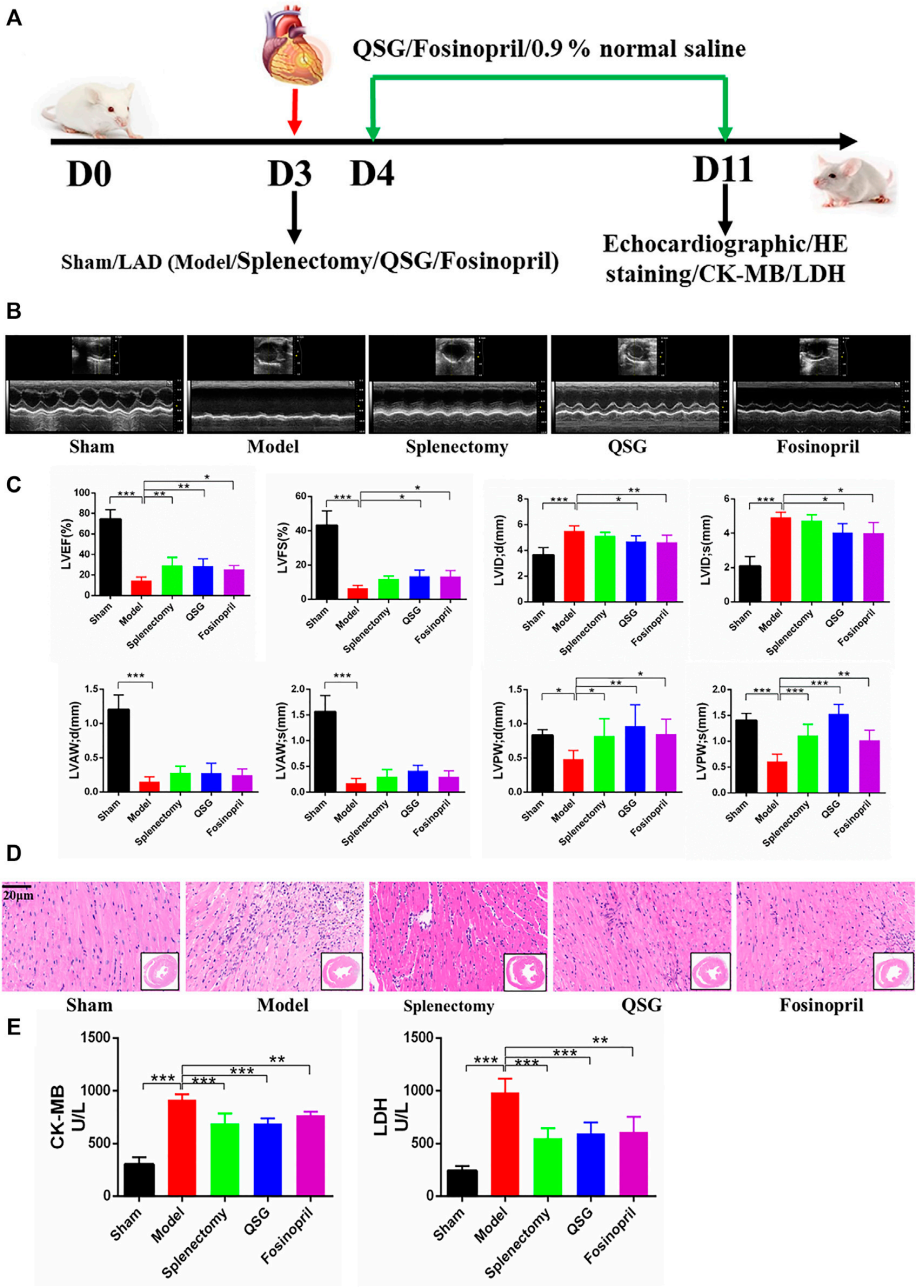
QSG improved cardiac function and reduced pathological changes in HF mice. **(A)** Experimental protocol for QSG studies in AMI-induced HF mice. **(B)** Representative M-mode echocardiographic images of mice in each group. **(C)** Evaluation of LVEF, LVFS, LVID; d, LVID; s, LVAW; d, LVAW; s, LVPW; d, LVPW; s levels in each group by echocardiography. N = 6 per group. **(D)** Representative images of HE staining in each group. Scale bar = 20 µm. **(E)** The level of CK-MB and LDH in serum from each group. N = 5 per group. *p < 0.05, **p < 0.01, ***p < 0.001 vs. model group.

Mice with AMI-induced HF were established, followed by treating with QSG, fosinopril or the same volume of 0.9% normal saline for 7 d. Echocardiography, HE staining, CK-MB and LDH detection were performed to determine whether QSG has a protective effect on cardiac function. Mice in the model group showed a significant reduction of cardiac function (Figures 1B,C), evidenced by the decrease of LVEF, LVFS, LVAW; d, LVAW; s, LVPW; d and LVPW; s (*p* < 0.001, *p* < 0.05) and increase of the LVID; d and LVID; s (*p* < 0.001), compared with the sham group. These variations of could be reversed by QSG, splenectomy and fosinopril group (*p* < 0.01; *p* < 0.05; *p* < 0.001). Further, HE staining showed that QSG could protect the heart from pathological changes and inflammatory cell infiltration (Figure 1D). Additionally, evidence suggested that QSG could inhibit the release of CK-MB and LDH into serum compared to the model group (Figure 1E) (*p* < 0.001). In summary, these data suggested that QSG could improve cardiac function in HF mice induced by LAD ligation.

### QSG Ameliorated the Pathological Changes of the Spleen by Inhibiting the Release of Splenic Monocytes

**FIGURE 2:**
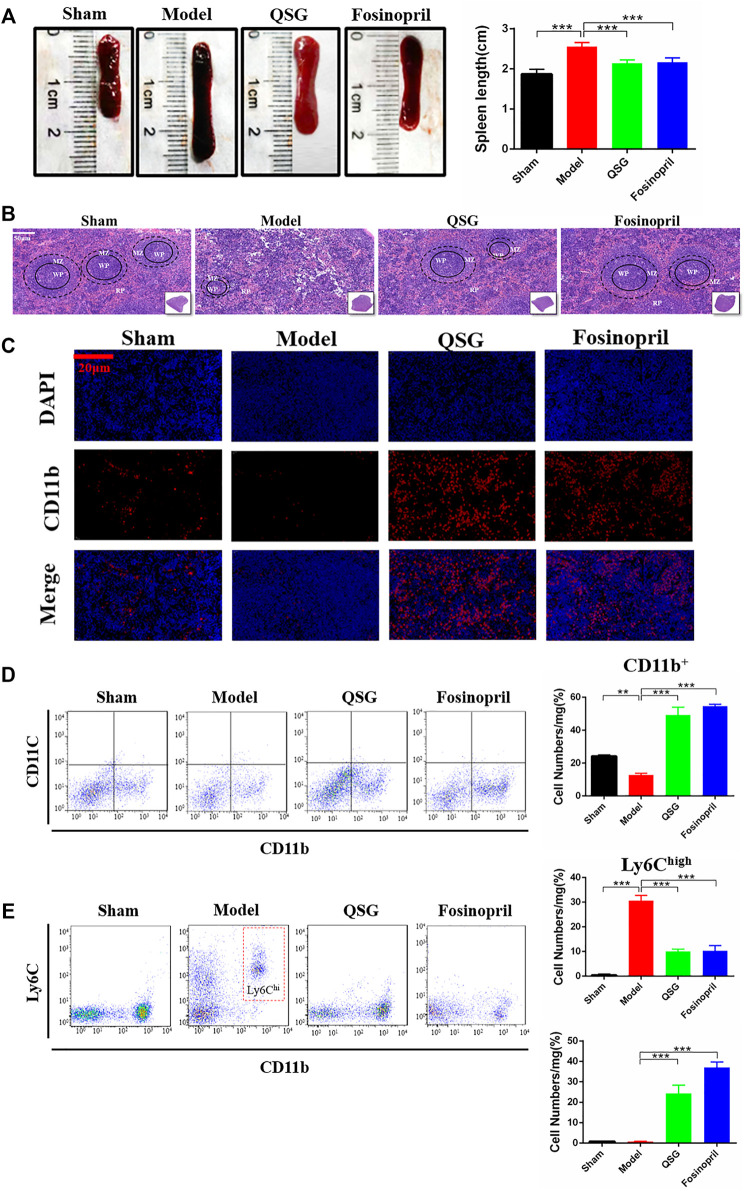
QSG inhibited the pathological changes of the spleen and the release of splenic monocytes in HF mice. **(A)** Representative photomicrograph of the spleen morphology and histogram of length statistics. N = 6 per group. **(B)** Representative spleen images of HE staining in each group. Scale bar = 50 µm. **(C)** Representative spleen tissue images of CD11b immunofluorescence from different groups. Scale bar = 20 µm. **(D)** Flow cytometry for CD11b monocytes counts and statistical analysis in the spleen. N = 3 per group. **(E)** Flow cytometry to detect the number of Ly6C^high^ and Ly6C^low^ monocytes in the spleen and statistical analysis. N = 3 per group. **p < 0.05, ***p < 0.001 vs. model group.

Morphological observation and flow cytometry analysis of the spleen were conducted in 7 d HF mice to further explore the relationship between the inflammatory cells infiltrated in the myocardial tissue and the variation of the spleen. Photographic observations showed increased spleen length and volume enlargement in the model group, while QSG was able to improve the spleen structure (Figure 2A). HE staining of the spleen tissue sections showed that the quantity of cells in the white marrow was reduced in the model group, the marginal area of the white pulp (WP) was blurred, and the number of red pulp (RP) under the envelope was decreased. Compared to the model group, QSG could reverse the structure of the WP marginal zone and increase the number of cells in the RP zone (Figure 2B). Especially QSG could outstanding inhibit the cell release of spleen in the WP. To further identify the cell type, CD11b was used to remark the monocytes. Immunofluorescence of spleen tissue showed that CD11b^+^ monocytes were markedly lower in the model group, while QSG could increase the number of CD11b^+^ monocytes (*p* < 0.001) (Figure 2C). In addition, results of flow cytometry for CD11b monocytes counts showed a significant decrease in the number of CD11b^+^ monocytes in the model group compared to sham group (*p* < 0.01), while there was a significant increase in the number of CD11b^+^ monocytes in the QSG group compared to the model group (*p* < 0.001) (Figure 2D). Further flow cytometry was performed to determine the ratio of CD11b^+^ monocytes differentiated into Ly6C^high^ and Ly6C^low^ in the spleen. The results showed that Ly6C^high^ monocytes in the model group were dramatically increased (*p* < 0.001), while Ly6C^low^ monocytes in the QSG group were increased compared to the model group (*p* < 0.001) (Figure 2E).

### QSG Regulated the Expression of Key Signaling Molecules in the TLR4-MyD88-NF-κB p65 Pathway in HF Mice

**FIGURE 3:**
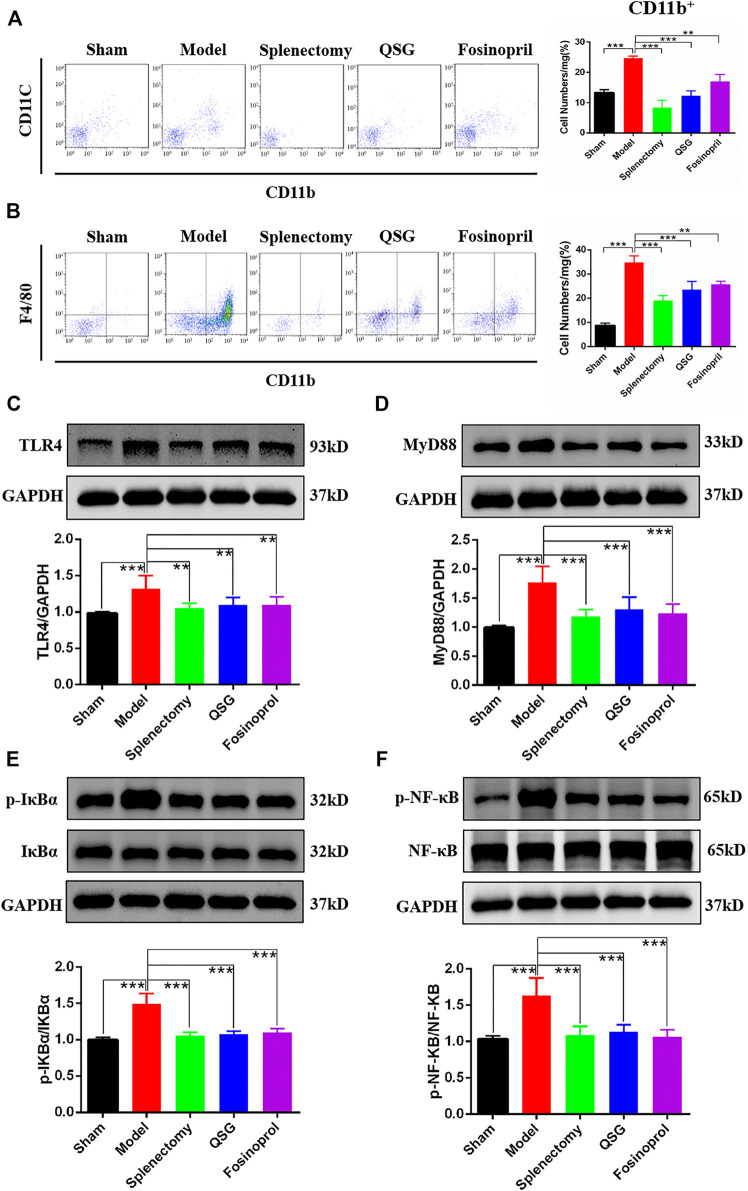
QSG exerted an anti-myocardial inflammation effect through the TLR4-MyD88-NF-κB p65 pathway in HF mice. **(A)** Flow cytometry detection of CD11b monocytes in blood and statistical analysis. N = 3 per group. **(B)** Flow cytometry detection of monocytes/macrophages in heart and statistical analysis. N = 3 per group. Representative western blot images and quantification of three experiments of TLR4 **(C)**, MyD88 **(D)**, p-IκBα **(E)**, and p-NF-κB p65 **(F)** in myocardial tissue. GAPDH was used as a loading control. N = 6 per group. **p < 0.01, ***p < 0.001 vs. model group.

The monocytes in the spleen can enter the blood circulation through the splenic vein and migrate to the myocardial tissue. The results of blood flow cytometry showed that CD11b^+^ monocytes in the model group were increased compared with the sham group (*p* < 0.001). Compared with the model group, CD11b^+^ monocytes in the QSG, splenectomy and fosinopril group were reduced (*p* < 0.001, *p* < 0.001, and *p* < 0.05, respectively) (Figure 3A). It suggested that QSG could inhibit the content of CD11b^+^ monocytes in blood circulation. CD11b^+^F4/80^+^ is often used as a method to detect the expression of macrophages in flow cytometry. The results showed that CD11b^+^F4/80^+^ macrophages in the heart from the model group were increased compared with the sham group. However, CD11b^+^F4/80^+^ macrophages in the heart from the QSG, splenectomy, and fosinopril group were reduced compared with the model group (*p* < 0.001, *p* < 0.001, and *p* < 0.05, respectively) (Figure 3B), suggesting that QSG could reduce the content of CD11b^+^F4/80^+^ in myocardial tissue.

Since TLR4 is a key receptor for damage-associated molecular patterns (DAMPs) stimulation on cardiomyocytes, myocardial tissue western blot analysis was used to explore the effect of QSG on the expressions of TLR4 and TLR4 pathway-related proteins. Results showed that compared with the sham group, the expression of TLR4 was increased in the model group, while it was decreased after QSG treatment (Figure 3C). Besides, as a critical pathway protein downstream of TLR4, MyD88 was upregulated in the model group compared with the sham group (Figure 3D). The increased phosphorylation level of IKBα (p-IKBα) and NF-κB p65 (p-NF-κB) indicated that as a key transcription factor in the inflammatory response, NF-κB p65 was activated, which could represent the level of inflammation to a certain extent. Further western blot results showed that p-IKBα and p-NF-κB p65 expression were elevated in the model group while reduced in the QSG and splenectomy groups (Figures 3E,F). In summary, these data suggested that QSG had a significant impact on the TLR4 pathway.

### QSG Inhibited the Inflammatory Response Induced by Macrophage Conditioned Medium in H9C2 Cells and Reduced Inflammatory Injury by Inhibiting TLR4-MyD88-NF-κB p65 Pathway

**FIGURE 4:**
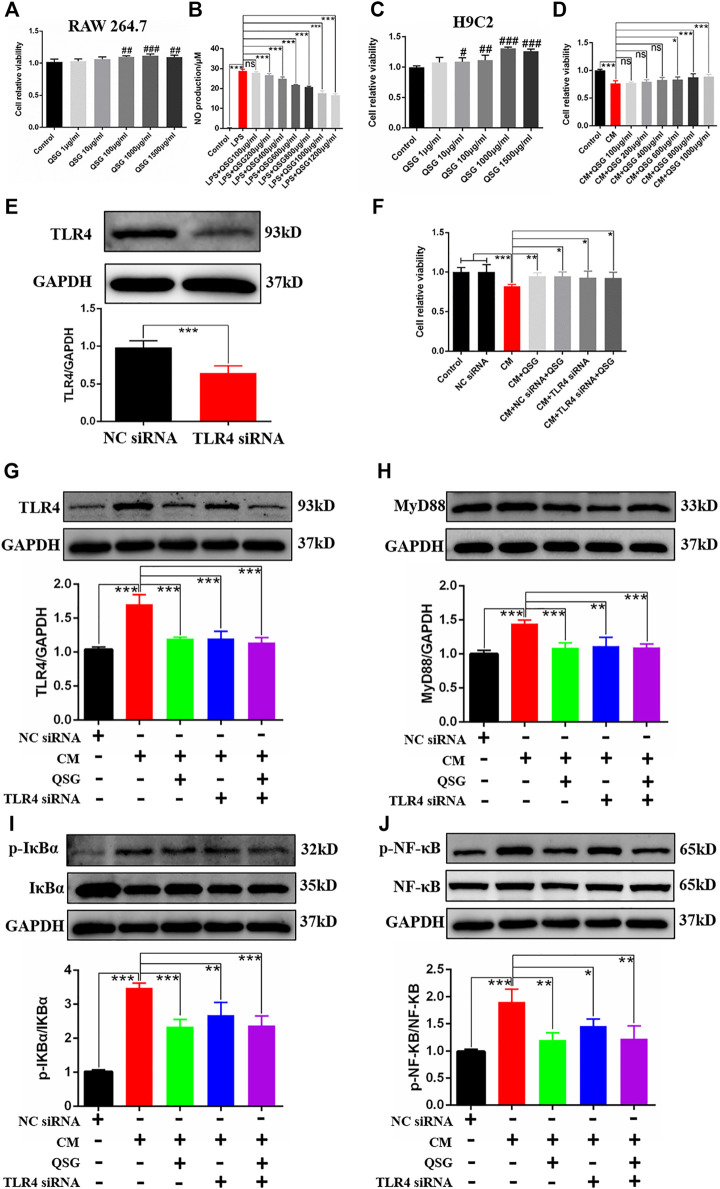
QSG might attenuate inflammatory injury in cardiomyocytes by inhibiting the TLR4-MyD88-NF-κB p65 pathway. **(A)** The CCK8 assay showed that QSG treatment for 24 h had no cytotoxic effect on RAW264.7 macrophages below 1,500 μg/ml, N = 6 per group. **(B)** The level of NO in the supernatant of RAW264.7 macrophages was evaluated by NO kit determination, N = 6 per group. **(C)** The CCK8 assay showed that QSG treatment for 24 h had no cytotoxic effect on H9C2 cells below 1,500 μg/ml, N = 6 per group. **(D)** The effective concentrations of QSG in CM-stimulated H9C2 cells were analyzed by CCK8 assay, N = 6 per group. **(E)** The knockdown efficiency of TLR4 siRNA in H9C2 cells was checked by western blot analysis, N = 3 per group. **(F)** TLR4 siRNA treatment increased the viability of CM-stimulated H9C2 cells. N = 6 per group. Representative western blot images and quantification of TLR4 **(G)**, MyD88 **(H)**, p-IκBα and IκBα **(I)**, p-NF-κB p65 and NF-κB p65 **(J)** in H9C2 cells from different groups, N = 3. GAPDH was used as a loading control. #*p* < 0.05, ^##^ *p* < 0.01, ^###^ *p* < 0.001 vs. control group. *p < 0.05, **p < 0.01, ***p < 0.001 vs. model group.

The specific mechanism of action of QSG in macrophages and cardiomyocytes, especially in myocardial inflammatory injury induced by macrophage, was revealed. Firstly, a model of LPS-induced RAW264.7 cells was established to investigate the anti-inflammatory effects of QSG on macrophages as previously described (Li et al., 2016). CCK8 results showed that QSG was non-toxic when co-treated with RAW264.7 cells below 1,500 μg/ml (Figure 4A). Meanwhile, QSG could reduce the amount of NO released into the cell supernatant from LPS-stimulated macrophages (Figure 4B). From these results, it was confirmed that QSG could inhibit the inflammatory activation of macrophages. Based on the CM-induced inflammatory H9C2 cell model established by the research group in the previous study (Wang X. et al., 2020), the anti-inflammatory effects of QSG on cardiomyocytes were further explored. The CCK8 results showed that co-treatment of H9C2 cells with QSG below 1,500 μg/ml was non-toxic (Figure 4C). Furthermore, there was a decrease in cell viability of H9C2 cells with CM stimulation (*p* < 0.001), while QSG at concentrations of 600–1,000 μg/ml could reverse this change (Figure 4D).

To validate whether QSG could exert anti-inflammatory effects in H9C2 cells through the TLR4 pathway, TLR4 siRNA was used to silence the expression of TLR4 as positive control (Figure 4E). Interestingly, silencing the expression of TLR4 could also increase the viability of CM-stimulated H9C2 cells as same as QSG treatment compared to the CM group (Figure 4F). Further western blot results showed that the expression of TLR4, MyD88, p-NF-κB p65, and p-IκBα was down-regulated after QSG and TLR4 siRNA treatment compared to CM-stimulated H9C2 cells (Figures 4G–J). Taken together, these results indicated that the TLR4 signaling cascade might be a potential target for QSG to combat inflammatory injury in cardiomyocytes.

### QSG Ameliorated Inflammatory Injury in Cardiomyocytes by Inhibiting the TLR4-MyD88-NF-κB p65 Pathway

**FIGURE 5:**
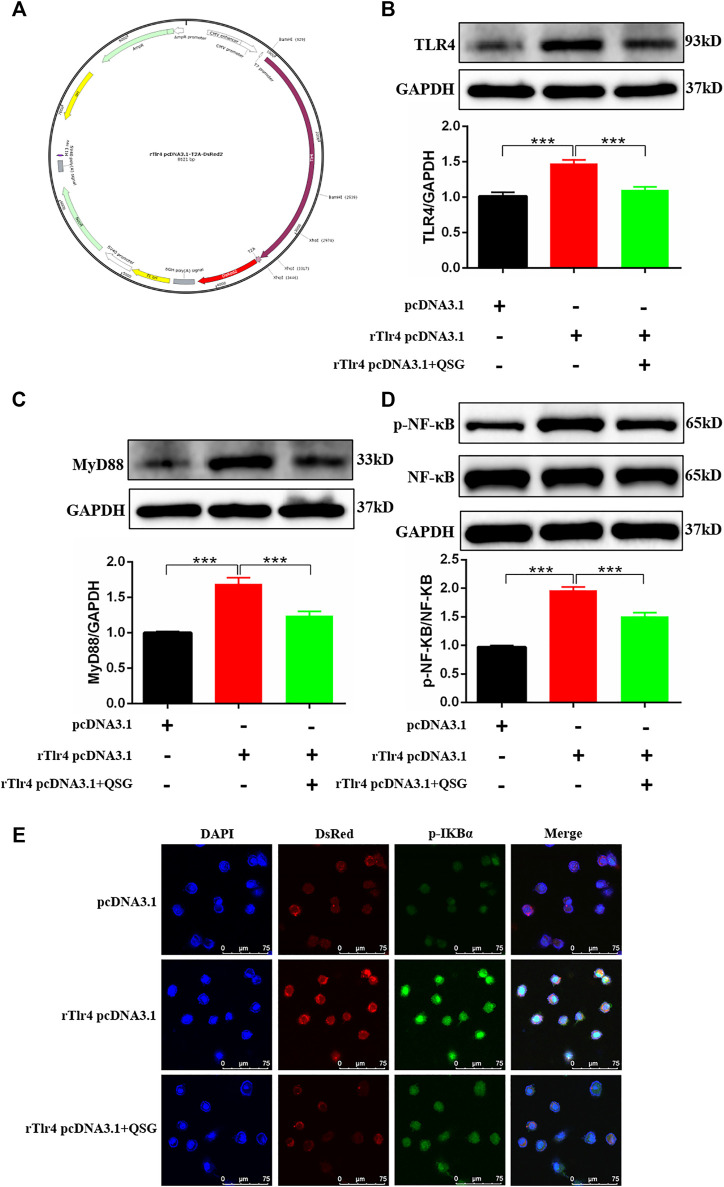
Validation of the regulatory effect by QSG on the TLR4-MyD88-NF-κB p65 pathway. **(A)** The structure of rTlr4 pcDNA3.1-T2A-DsRed plasmid. Representative western blot images and quantification of TLR4 **(B)**, MyD88 **(C)**, p-NF-κB p65 and NF-κB p65 **(D)** in H9C2 cells, N = 3 per group. GAPDH was used as a loading control. *p < 0.05, **p < 0.01, ***p < 0.001 vs. rTlr4 pcDNA3.1-T2A-DsRed transfected group. **(E)** Representative immunofluorescence staining images of p-IκBα and DsRed from different groups. H9C2 cells were transfected with rTlr4 pcDNA3.1-T2A-DsRed to check the overexpression of TLR4. pcDNA3.1-T2A-DsRed transfected cells were used as a negative control. *p < 0.05, **p < 0.01, ***p < 0.001 vs. rTlr4 pcDNA3.1-T2A-DsRed transfected group.

TLR4 was overexpressed in H9C2 cells by transfecting rTlr4 pcDNA3.1-T2A-DsRed plasmid to further validate whether TLR4 is a key target for QSG to regulate the inflammatory response of cardiomyocytes (Figure 5A). Western blot results indicated successful overexpression of TLR4 in H9C2 cells (Figure 5B). Meanwhile, downstream inflammatory effector molecules, MyD88 and p-NF-κB p65, were activated after overexpression of TLR4 in cardiomyocytes (Figure 5C,D). Besides, compared with the rTlr4 pcDNA3.1-T2A-DsRed group, QSG can inhibit the expression of TLR4, MyD88, and p-NF-κB p65 (Figures 5B–D). Furthermore, immunofluorescence results showed that compared with cells transfected with pcDNA3.1-T2A-DsRed, TLR4 overexpression increased the expression of p-IκBα, representing the activation of p-NF-κB p65 (Figure 5E). The lower red fluorescence (DsRed) in the figure indicated a decrease in exogenous TLR4 expression in QSG-treated cells. In conclusion, these data suggest that QSG can act on the TLR4-MyD88-NF-κB p65 pathway to function.

## Discussion

Previous studies had suggested that QSG could modulate the phenotype of macrophages in the heart, thereby inhibiting myocardial fibrosis and promoting angiogenesis (Lu et al., 2019). However, how QSG regulates the release of monocytes in the spleen and the phenotype-mediated myocardial inflammation mechanism has not yet been revealed. The current findings were summarized as follows: 1) QSG improved cardiac function and reduced pathological changes in HF mice. 2) QSG ameliorated the pathological changes of the spleen, inhibited the release of splenic monocytes, and reduced the recruitment of macrophages to the heart. 3) QSG exerted an anti-myocardial inflammation effect through the TLR4-MyD88-NF-κB p65 pathway in HF mice. 4) QSG reduced inflammation injury through the TLR4-MyD88-NF-κB p65 pathway in CM-induced H9C2 cells.

HF is a serious end-stage of many cardiovascular diseases and can be caused by an inflammatory response (Schefold et al., 2016; Michels da Silva et al., 2019; Peet et al., 2020). In terms of the pathological mechanism of HF, the inflammatory response has been proven to be an important key event in the induction of acute myocardial infarction and is related to macrophage infiltration (Glezeva and Baugh, 2014; Kingery et al., 2017; Louwe et al., 2020). Previous studies have reported that when AMI occurs, monocytes derived from the spleen enter the blood and are recruited to the myocardial injury area in two stages. The first stage is mainly Ly6C^high^ monocytes, and the second stage is mainly Ly6C^low^ monocytes (van der Laan et al., 2014; Fernández-García et al., 2020). However, fewer Ly6C^low^ monocytes were recruited to the infarct area than early recruitment of Ly6C^high^ monocytes. Interestingly, Ly6C^high^ monocytes could give rise to Ly6C^low^ monocytes in the late healing state (Dutta and Nahrendorf, 2015). Ly6C^high^ monocytes produce early inflammatory macrophages (M1), while Ly6C^low^ monocytes differentiate into anti-inflammatory macrophages (M2) to play a role in tissue repair and angiogenesis. Therefore, effective regulation of the release of splenic monocytes could greatly inhibit the dramatic decline in heart function during HF. In this study, LAD ligation and splenectomy were used to explore the role of splenic monocytes in HF. The results showed that consistent with the splenectomy group, QSG was able to inhibit morphological changes, monocyte release, and phenotypic transformation in the spleen. Moreover, it could also inhibit macrophage recruitment and activation in the myocardial injury area. Finally, QSG could improve cardiac function and delay the progression of HF.

TLRs are well-studied pattern recognition receptors in the body’s autoimmune system and play an essential role in the pathology of autoimmune diseases, acute and chronic inflammation, and cardiovascular diseases (Jaén et al., 2020; McKernan, 2020; Su et al., 2020; Li et al., 2021). As a member of the TLR family, TLR4 is widely found on the surface of macrophages, cardiomyocytes, and other cells (Han et al., 2017; Wang Y. et al., 2020; Tang et al., 2020). TLR4 could induce the production of inflammatory factors by recruiting MyD88, thereby activating MyD88-dependent signaling pathways (Firmal et al., 2020). As a signaling pathway downstream of MyD88, NF-κB p65 is a key transcription factor in the inflammatory response (Yan et al., 2020). NF-κB is presented in the cytoplasm as an inactive IκB/NF-κB complex in the resting state, whereas it could promote gene transcription of cytokines, chemokines, and inflammatory factors, and amplify inflammatory signals after activation (Hoffmann et al., 2002). Therefore, the regulation of the TLR4 pathway offers new possibilities for HF prevention and treatment.

Whether QSG could exert anti-inflammatory and cardioprotective effects through the TLR4 signaling pathway was further determined. Western blot analysis showed that QSG administration could downregulate the levels of TLR4, MyD88, p-IκBα, and p-NF-κB p65 *in vivo*, suggesting that the anti-inflammatory effects of QSG might act through the TLR4-MyD88-NF-κB p65 pathway. Furthermore, a CM model of inflammation induced by LPS-stimulated macrophages in H9C2 cells was used combined with TLR4 siRNA or rTlr4 pcDNA3.1-T2A-DsRed plasmid to knockdown/overexpress TLR4 gene expression *in vitro* study. Results indicated that, like knockdown of TLR4, QSG could also attenuate inflammatory injury in H9C2 cardiomyocytes and reduce the expression of proteins in the TLR4 signaling pathway. Moreover, when TLR4 was overexpressed, QSG could still reduce the expression of TLR4 and the protein levels of MyD88, p-IκBα, and p-NF-κB p65 in downstream pathways. The above results demonstrated that QSG could play a cardioprotective effect via the TLR4-MyD88-NF-κB p65 pathway.

## Conclusion

**FIGURE 6:**
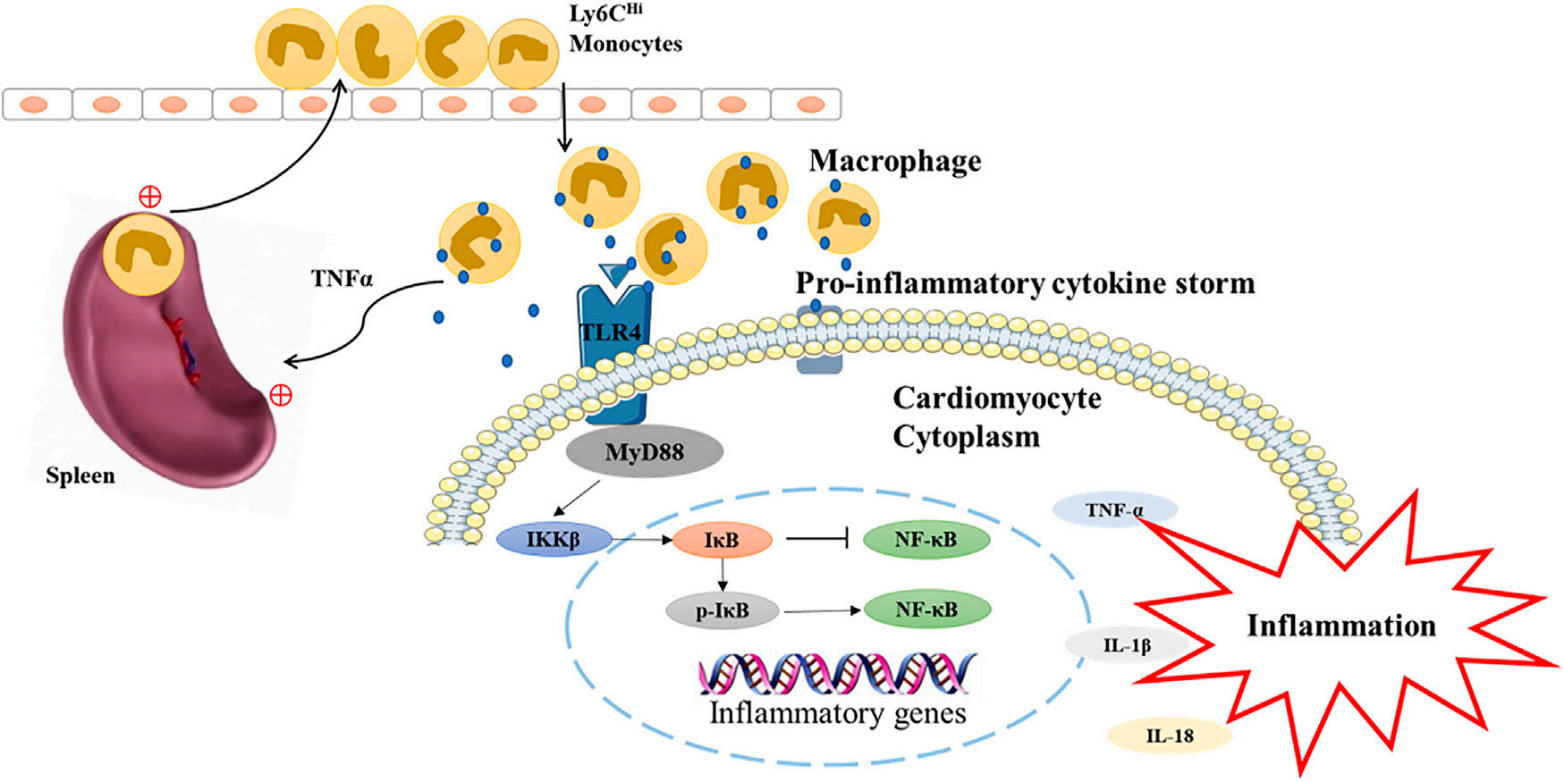
The underlying mechanism of QSG in the treatment of heart failure is mediated by TLR4-MyD88-NF-κB p65 pathway.

This study demonstrated QSG could improve cardiac function and reduce the inflammatory response in AMI-induced HF by inhibiting splenic monocyte release and protecting myocardial function via the TLR4-MyD88-NF-κB p65 pathway in heart failure mice (Figure 6). The current study provides a clue that QSG can be used as an alternative therapeutic strategy for anti-AMI-induced HF. However, we currently lack a more in-depth mechanistic exploration of the cardio-splenic axis. The relationship between splenic monocytes and heart failure will be further investigated on this basis at a later stage.

## Data Availability

The original contributions presented in the study are included in the article/Supplementary Material, further inquiries can be directed to the corresponding authors.
